# NGLY1 Deficiency Zebrafish Model Manifests Abnormalities of the Nervous and Musculoskeletal Systems

**DOI:** 10.3389/fcell.2022.902969

**Published:** 2022-06-13

**Authors:** Aviv Mesika, Golan Nadav, Chen Shochat, Limor Kalfon, Karen Jackson, Ayat Khalaileh, David Karasik, Tzipora C. Falik-Zaccai

**Affiliations:** ^1^ Institute of Human Genetics, Galilee Medical Center, Nahariya, Israel; ^2^ Azrieli Faculty of Medicine, Bar Ilan University, Safed, Israel; ^3^ MIGAL, Galilee Research Institute, Kiryat Shmona, Israel

**Keywords:** NGLY1 deficiency, zebrafish, nervous system, musculoskeletal system, abnormalities

## Abstract

**Background:** NGLY1 is an enigmatic enzyme with multiple functions across a wide range of species. In humans, pathogenic genetic variants in *NGLY1* are linked to a variable phenotype of global neurological dysfunction, abnormal tear production, and liver disease presenting the rare autosomal recessive disorder N-glycanase deficiency. We have ascertained four NGLY1 deficiency patients who were found to carry a homozygous nonsense variant (c.1294G > T, p.Glu432*) in *NGLY1*.

**Methods:** We created an *ngly1* deficiency zebrafish model and studied the nervous and musculoskeletal (MSK) systems to further characterize the phenotypes and pathophysiology of the disease.

**Results:** Nervous system morphology analysis has shown significant loss of axon fibers in the peripheral nervous system. In addition, we found muscle structure abnormality of the mutant fish. Locomotion behavior analysis has shown hypersensitivity of the larval *ngly1* ^(*−/−*)^ fish during stress conditions.

**Conclusion:** This first reported NGLY1 deficiency zebrafish model might add to our understanding of NGLY1 role in the development of the nervous and MSK systems. Moreover, it might elucidate the natural history of the disease and be used as a platform for the development of novel therapies.

## 1 Introduction

N-glycanase deficiency (OMIM **#** 615273) is a rare autosomal recessive disorder linked to a variable phenotype in the patients (Enns et al., 2014) and caused by pathogenic variants in *NGLY1* (Need et al., 2012; Lam et al., 2017). Currently, there are fewer than 100 patients reported worldwide, and possibly many more undiagnosed, who suffer from this devastating disease with no effective treatment (Tickotsky-Moskovitz, 2015). One of the major manifestations in patients with N-glycanase deficiency is severe global developmental delay (motor and cognitive), neurological dysfunction and damage to both the central nervous system (CNS) and peripheral nervous system (PNS), leading to significant locomotion disability (Jones et al., 2012). Furthermore, early onset liver disease and elevated liver transaminases are also part of the clinical features in the disorder. Finally, the most unique phenotype of N-glycanase deficiency is abnormal tear production, evident as absence of tears (alacrima).

NGLY1, also known as peptide:N-glycanase (PNGase), is an enzyme with versatile functions across a wide range of species encoded by *NGLY1* gene (Suzuki, 2015). NGLY1 is one of the crucial cytosolic proteins involved in ERAD pathway. Its specific function, shown in yeast, is to catalyze glycoprotein de-glycosylation by cleaving the aspartyl glycosylamine bond of N-linked glycoproteins (Suzuki et al., 2016). NGLY1 was first described by Suzuki et al. who identified a specific signaling pathway, that is, disrupted in the absence of *Pngl* (the orthologue of ngly1) enzyme in *Drosophila* (Galeone et al., 2017). Additional evidence for developmental function of *NGLY1* was shown in *Caenorhabditis elegans* (*C. elegans*), where its orthologue has a developmental role in the regulation of neuronal branching development during organ innervations (Habibi-Babadi et al., 2010). Kong et al. (2018) reported that N-glycanase deficiency impairs mitochondrial physiology in diverse invertebrate and vertebrate species such as *C. elegans*, mice and humans. In 2017, Tomlin et al. (2017) revealed that the cytosolic enzyme NGLY1 is essential for Nuclear Factor, Erythroid 2 Like 1 (Nrf1) activation in response to proteasome inhibition. Recently, NGLY1 was shown to have a crucial role in cell homeostasis as it regulates cell volume by adjusting the amount of water channeled (aquaporins) to the membrane surface in response to environmental changes such as hypotonic/hypertonic conditions (Tambe et al., 2019), evidence that may be relevant to the phenotype of alacrima in patients. Recently, various animal models have been established for NGLY1 deficiency, including mouse (Fujihira et al., 2017; Asahina et al., 2021), rat (Asahina et al., 2020) and *Drosophila* (Galeone et al., 2017), but each different model has displayed different phenotypes that provide partial understanding of the pathophysiology underlying the disease. Zebrafish (*Danio rerio*) have emerged in recent years as a major model organism for biomedical research and have unique advantages for the research of rare genetic diseases (Lieschke and Currie, 2007). Zebrafish model allows mimicking of human disease (Vaz et al., 2019) due to high homology of anatomical structures and the fact that zebrafish share the same organs and biological systems with humans (White et al., 2013; Varga et al., 2018). We established a novel animal model for NGLY1 deficiency using zebrafish, which carry one copy of the gene (https://zfin.org/ZDB-GENE-050522-535#summary). We studied its phenotype and confirmed that *ngly1* mutant fish develop neurological and muscle pathology. The zebrafish model might add to the understanding of NGLY1 role in development and maintenance of the nervous (Levitas-Djerbi and Appelbaum, 2017) and MSK systems (Mignani et al., 2020). Moreover, developing this novel fish model might serve as an excellent platform for testing possible novel therapies for this devastating, untreatable disease (Dinday and Baraban, 2015; Basnet et al., 2019).

## 2 Materials and Methods

### 2.1 Clinical Data

The patients were recently reported (Kalfon et al., 2021). The guardians of the affected individuals signed an informed consent form for participating in this study. The Israeli Supreme Helsinki committee approved the study; GMC 03-04-2006.

### 2.2 Cell Culture

Primary fibroblast cell cultures were derived from skin biopsies taken from one NGLY1 deficiency patients and maintained in a 37°C and 5% CO_2_ incubator.

### 2.3 Western Blotting

Proteins were extracted from fibroblast cells of healthy donors and affected individuals as well as from amputated fish tails (3mpf) of WT and mutant fish. Electro-blotting was done using a power supply with the conditions of 60 V/180 mA, for 1.5 h. NGLY1 primary antibody, at 1:500 concentrations, was added to the membrane for overnight incubation at 4°C, and then underwent 1 h of incubation with horseradish peroxidase (HRP) secondary antibody *Goat-anti-rabbit* at 1:2000 concentration. Finally, the membrane was filmed using G:BOX (Syngene, Frederick, WA, United States) with a chemiluminescence light.

#### 2.3.1 Antibodies Used

Anti NGLY1 antibody (Rabbit anti Human), Sigma-Aldrich, HPA036825 (Sigma-Aldrich, St Louis, MO, United States). Secondary antibody IgG-HRP Goat anti-Rabbit, Santa Cruz Biotechnology (Dallas, Texas United States; Cat. No. SC 2004), Anti β-Actin antibody, anti-Mouse monoclonal, cell-signaling technology (Danvers, MA, United States) Cat. No. 8H10D10, Secondary antibody, IgG-HRP Goat anti-Mouse, Invitrogen (Paisley, United Kingdom; Cat. No. A16072).

### 2.4 Immunofluorescence Staining

The cells were fixed with 4% formaldehyde, permeabilized with 0.5% *Triton X-100* in PBS, and blocked with blocking solution (10% FBS, 1% BSA, and 0.05% Triton X-100 in PBS). Immunofluorescence staining was performed with 1:100 Anti NGLY1 antibody (Rabbit anti-Human), Sigma-Aldrich, HPA036825 in blocking solution overnight at 4°C. Next, the cells were incubated with 1:700 Alexa 488-labeled secondary antibody (A-21202 by Invitrogen, excitation at ∼495 nm, emission ∼519 nm). Finally, cover slips were laid on slides containing 4 µl of mounting medium with DAPI nucleus staining (Vector Laboratories, Burlingame, CA, United States). Fluorescent images were obtained by Nikon Eclipse Ti and Nikon C2 confocal scanner (Nikon Instruments Inc., Melville, NY, United States). Fluorescence images were analyzed with ImageJ software.

### 2.5 Bioinformatic Tools and Online Databases

Bioinformatic software and databases were used throughout the research, as follows:

Primer design tools: PRIMER3, NCBI primer BLAST.

Genome databases: UCSC, ENSEMBLE genome browser, NCBI PUBMED, OMIM (Online Mendelian Inheritance in Man), GeneCards, HGMD (Human Genome Mutation Database), dbSNP (Database of Single Nucleotide Polymorphism).

Analysis tools: Nucleotide primer BLAST, reverse complement tool, Mutation Taster, Alamut Visual. Restriction enzymes design tools: NEB Cutter. CRISPR design (fish) tool https://crispr.cos.uni-heidelberg.de/.

### 2.6 Generating Zebrafish Model

All animal research followed a protocol approved by BIU IACUC- 023_b15280_80.

### 2.7 Ngly1 Mutant Zebrafish Established by Targeting Ngly1 Exon 9 Using CRISPR-Cas9

In order to create a premature stop codon in exon 9 similar to the genetic variant in our NGLY1 patients, we used CRISPR/Cas9 mediated *ngly1* targeting in ZF embryo (Figure 2A). A guide RNA (gRNA) was designed to target exon 9 of *ngly1* (NM_001020601.1). We analyzed *ngly1* CRISPR/Cas9 mediated gene targeting in G0 generation by extracting DNA from 24hpf injected embryos to determine mutagenesis efficiency. Cas9 protein and gRNAs (Sigma-Aldrich) targeting *ngly1* exon 9 were injected to one cell stage zebrafish embryo. Twenty-four hours after injection a sample of injected embryos was genotyped by PciI restriction enzyme which has a recognition site at the Cas9 nuclease cleavage site. In order to obtain *ngly1* knockout zebrafish, F0 founder fish harboring *ngly1* mutation were mated with WT. F1 heterozygotes (3mpf) were genotyped. Two different *ngly1* mutant alleles were found (Table 1; Figure 2B). We crossed F1 del 19bp fish with a desired wild-type (WT) fish (outcrossing) in order to reduce off-target mutations effect. F2 heterozygotes were crossed with each other to create *ngly1* ^(*−/−*)^ and *ngly1* ^(*+/+*)^ fish.

**TABLE 1:** Heterozygous fish with *ngly1* mutant alleles.

Fish number	Allele 1		Allele 2	
	Nucleotide change	Amino acid change	Nucleotide change	Amino acid change
1	c.1759-1777del	p.517fs	**-**	**-**
2	c.1553-1557del	p.443fs	**-**	**-**

### 2.8 Zebrafish Maintenance

Zebrafish (*Danio rerio*) of AB strain were maintained at 28°C under 14 h light: 10 h dark cycles. For the experiments, adults and larval fish were euthanized by emersion in methane sulfonate (MS222), Tricaine 0.4% with subsequent placing on ice.

### 2.9 The Guide RNA Design

The website (https://crispr.cos.uni-heidelberg.de/) was used to design gRNAs for targeting exon 9 of zebrafish *ngly1* gene. The gRNA designed contained recognition site for PciI restriction enzyme near the PAM sequence which allows screening for CRISPR`s success in mutating exon 9 of *ngly1*.

### 2.10 Embryo Injection

A mix of *ngly1* gRNA and Cas9 mRNA was injected directly into one-cell-stage embryos, using a pneumatic Pico Pump (WPI, Worcester, MA, United States). Each embryo was injected with 500 pl of solution containing ∼300 ng/μl of gRNA and ∼300 ng/μl of Cas9 protein.

### 2.11 Zebrafish Genotyping

#### 2.11.1 Tail Clipping (Adults)

Fish at 3 months post fertilization (*mpf*) were anesthetized by immersion in fish system’s water containing 0.016 mg/ml Tricaine (Sigma-Aldrich) and tail clipping was performed.

#### 2.11.2 DNA Extraction From zebrafish Tail and PCR Amplification

DNA was extracted from fish tail (3mpf) by using a standard protocol.

### 2.12 cDNA Synthesis

#### 2.12.1 RNA Purification From ZF Tail

RNA and cDNA production was performed from fish tail (3mpf) using standard protocol.

#### 2.12.2 mRNA Levels Evaluation

cDNA amplicons for *ngly1* and *rpl32* were evaluated using RT-PCR. *rpl32* was used as a reference gene and normalized to healthy controls.

#### 2.12.3 Fluorescent *In-Situ* Hybridization

Fluorescent probe was designed in order to label the *ngly1* mRNA in zebrafish larvae tissues (Fried and Sherma, 2010) (Integrated DNA Technologies, Coralville, IA, United States). At 6*dpf* the larvae were fixed in 4% paraformaldehyde (Sigma-Aldrich). Next the larvae were dehydrated with 25%, 50%, and 75% ethanol for 15 min each. Larvae were incubated with 10 μg/ml proteinase K for 45 min to elevate the permeability of the tissues. Finally, the larvae were incubated with 200 ng/μl *ngly1* probe: (5′CCG​TCT​ACC​ACA​TAG​ACC​GTG​CTT​GTC​TCC​CGC​GAA​GGA​AAC​CCT​CCT​ACT​CGA​CCT​TCA​AGT​TGA​CGC​GAG​GTC​GTC​CT-3′) and visualized under Nikon Eclipse Ti and Nikon C2 confocal scanner (Nikon Instruments Inc.). In order to validate the *ngly1* expression in the nervous system.

### 2.13 Establishing a Stable Ngly1 Mutant Transgenic Zebrafish Line

Tg (HUC:gal4 uas:mem YFP-mito CFP) co-expresses memYFP and mitoCFP (Zada et al., 2014) and was kindly provided by Prof. Lior Appelbaum (Bar-Ilan University, Ramat-Gan, Israel). In order to establish a *ngly*1 mutant transgenic fish we crossed F0 Tg (HUC:gal4 uas:mem YFP-mito CFP) to homozygote *ngly1* ^(*−/−*)^ and selected heterozygotes from F1. F1 heterozygotes whose in-breeding produced homozygous *ngly1* ^(*−/−*)^/Tg (HUC:gal4 uas:mem YFP-mito CFP) and *ngly1* ^(*+/+*)^/Tg (HUC:gal4 uas:mem YFP-mito CFP) siblings (F2). The PNS morphology was imaged by confocal Nikon eclipse.

### 2.14 Phalloidin Staining

Larvae at 6*dpf* were fixed in 4% paraformaldehyde (Sigma-Aldrich). Next the larvae were dehydrated with 50% and 70% ethanol and then stained with Phalloidin fluorescent dye that stains F-actin in fixed cells, stabilizes actin filaments *in vitro* and visualizes them (Wulf et al., 1979). Finally, we focused on larval muscle by visualization under fluorescent microscope- Nikon Eclipse Ti and Nikon C2 confocal scanner (Nikon Instruments Inc.). Images were converted in 12 bit using software and exported in *tiff* format. Fluorescence was measured using Nikon Eclipse Ti software; data obtained were represented as the ratio between total pixel intensity and the number of pixels. Mean size of the somite was measured in µm using Nikon Eclipse Ti software. Muscle morphology and intensity were compared between *ngly1* ^(*−/−*)^ and *ngly1* ^(*+/+*)^ siblings (Mignani et al., 2020).

### 2.15 Motor Assay

Zebrafish larvae at 6*dpf* were placed in 96-wells plate, and behavior was recorded by DanioVision (Noldus Information technology, Wageningen, Netherlands) system. The results were analyzed by the Ethovision XT -11 locomotion tracking software (Noldus). We performed a basic locomotor assay that consisted of three different phases: 15 min lights-on, then 15 min lights-off (stress stimulus) and finally 15 min of lights-on (recovery). *ngly1* ^(*−/−*)^ and *ngly1* ^(*+/+*)^ siblings were video tracked, and swimming distance patterns were measured and analyzed.

### 2.16 Pain Sensitivity Test

A pain sensitivity test based on sensitized acid aversion assay developed by Steenbergen and Bardine (2014) was performed; the premise of this experiment is based on the increase in movement presented by larvae exposed to acid environment. 6*dpf* zebrafish larvae were placed in 96 wells plate containing an insert of 96 wells with a 100-um mesh bottom (Millipore, Massachusetts United States); all larvae could be simultaneously moved from the water with a neutral pH to acetic acid 0.01% water (pH = 3.7–3.9) where they stayed for 10 min. Fish behavior was recorded by DanioVision (Noldus) system for 10 min, and swimming distance patterns of *ngly1* ^(*−/−*)^ and *ngly1* ^(*+/+*)^ siblings were calculated.

## 3 Results

### 3.1 Fibroblast Cell Culture Analysis

**FIGURE 1:**
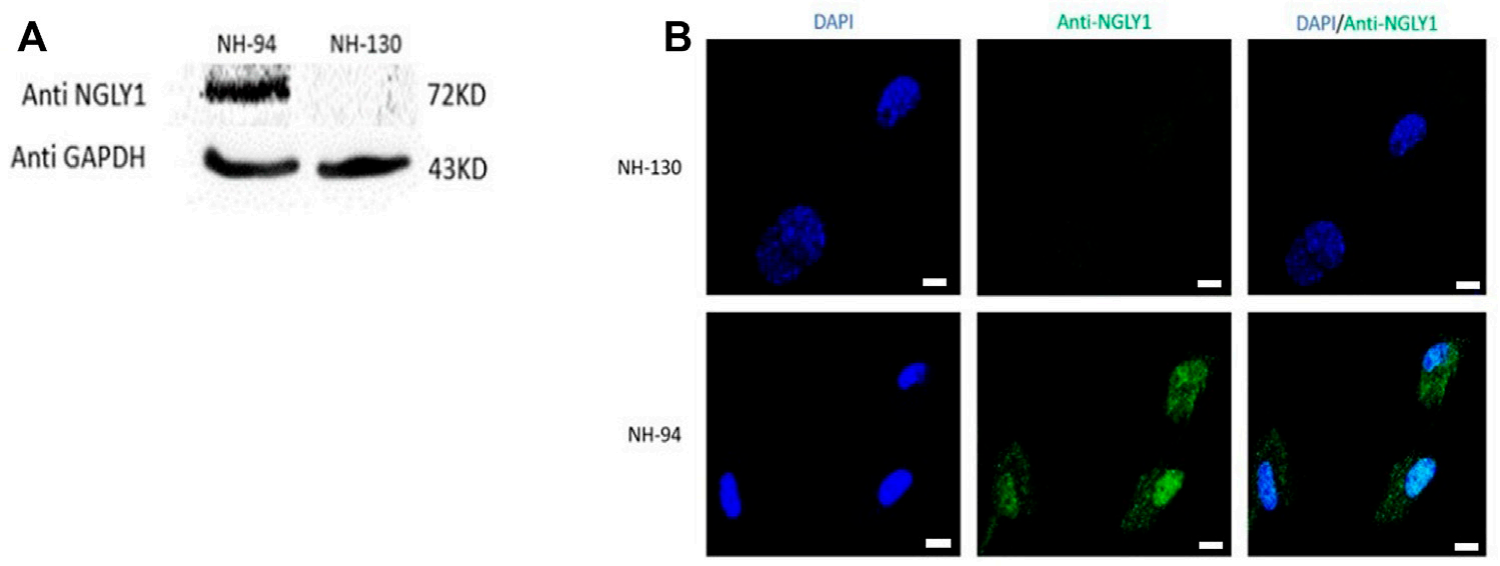
NGLY1 protein levels are reduced in patients with NGLY1 deficiency. **(A)** WB analysis of NGLY1 protein with an antibody against human NGLY1, GAPDH was used as a loading control. **(B)** Immunofluorescence staining of NGLY1 protein level. Blue: DAPI, green: anti-NGLY1. NH-94 = healthy control; NH-130 = Patient. Scale bars- 20 µm.

In order to investigate the studied genetic variant effect on NGLY1 protein level in humans, proteins were extracted from fibroblasts derived from a patient (NH-130) and a healthy control (NH-94). NGLY1 protein was absent in the patient with NGLY1 deficiency (Figures 1A,B).

### 3.2 Zebrafish mRNA and Protein Expression

**FIGURE 2:**
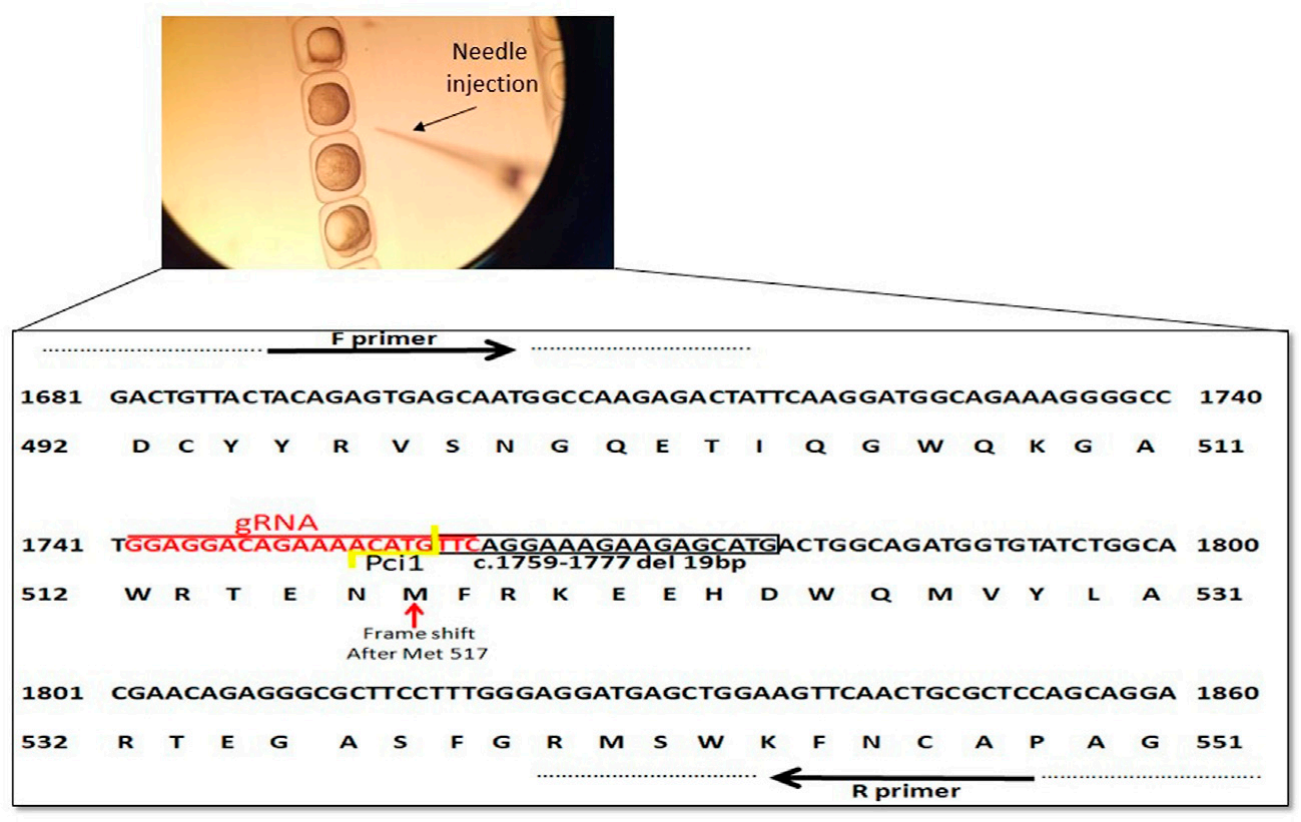
Schematic representation of *ngly1* gene targeting in zebrafish. CRISPR/Cas9 gRNA in exon9 (in red), deletion of 19bp (c.1759–1777) is marked by black box and frameshift starting point is indicated by red arrow. Primers used for PCR amplification are marked.

**FIGURE 3:**
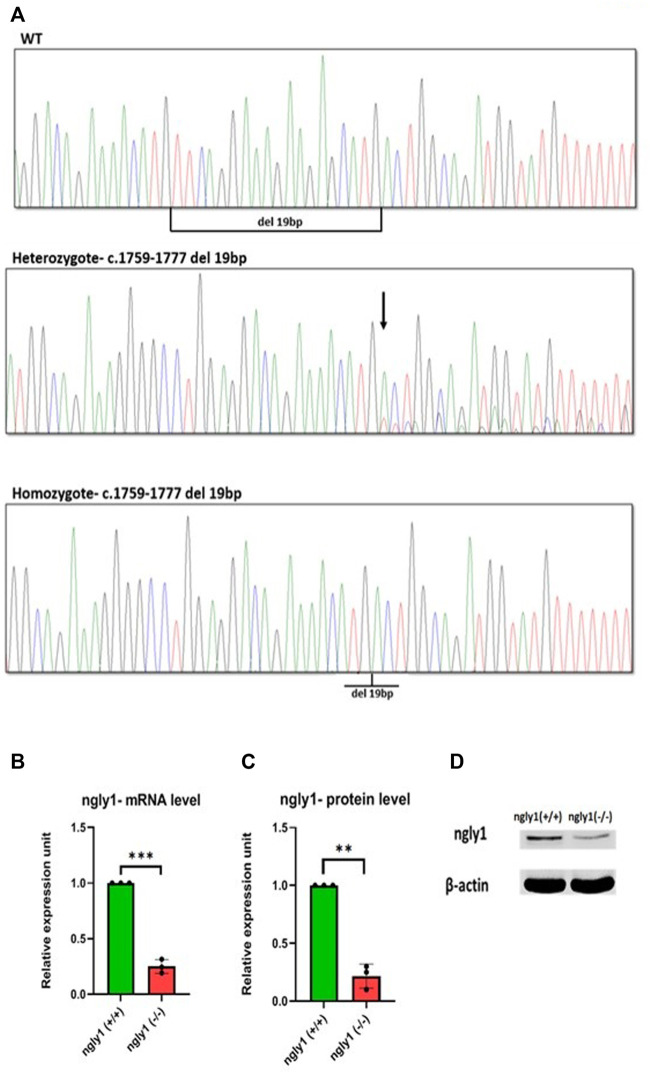
Mutation characterization in zebrafish**- (A)** Genotype screening in DNA extracted from zebrafish, Representative electrophoregrams indicating the sequences of WT, heterozygote and homozygote. **(B)** qPCR analysis quantification of ngly1 mRNA levels. t-test (*ngly1* ^(*+/+*)^ *n* = 3, *ngly1* ^(*−/−*)^ *n* = 3, ***p < 0.001). **(C)** WB analysis quantification of ngly1 protein levels. t-test (*ngly1* ^(*+/+*)^ *n* = 3, *ngly1* ^(*−/−*)^ *n* = 3, **p < 0.01). **(D)** WB analysis with an antibody against ZF ngly1; β-actin was used as a loading control.

A high percentage (>90%) of embryos harbored mutations at *ngly1* exon 9, indicating the efficiency of CRISPR-Cas9 in inducing mutations with the *ngly1* gRNA designed. Out of 62 F1, 14 fish were heterozygotes to a deletion of 19bp (c.1759–1777) resulting in p.met 517fs (Figures 2A,B). This mutation causes the appearance of a premature stop codon creating a 535 amino acid protein instead of the 644 amino acid full length of ngly1 zebrafish protein. The 14 F1 heterozygote fish were crossbred with each other to obtain the F2 generation. F2 were in-bred to generate F3 *ngly1* homozygote, heterozygote and WT offspring for phenotype-genotype correlations analysis (Figure 3A). A significantly lower *ngly1* mRNA expression was detected in the *ngly1* ^(*−/−*)^ fish compared to the *ngly1* ^(*+/+*)^ fish as expected (*p* < 0.001) (Figure 3B). The positive control *rpl32* mRNA showed similar expression levels in both genotypes. Similarly, the western blot analysis demonstrated a significant reduction in protein level of ngly1 in the *ngly1* ^(*−/−*)^ fish compared to the *ngly1* ^(*+/+*)^ fish (*p* < 0.01) (Figures 3C,D).

### 3.3 Ngly1 Expression Pattern

**FIGURE 4:**
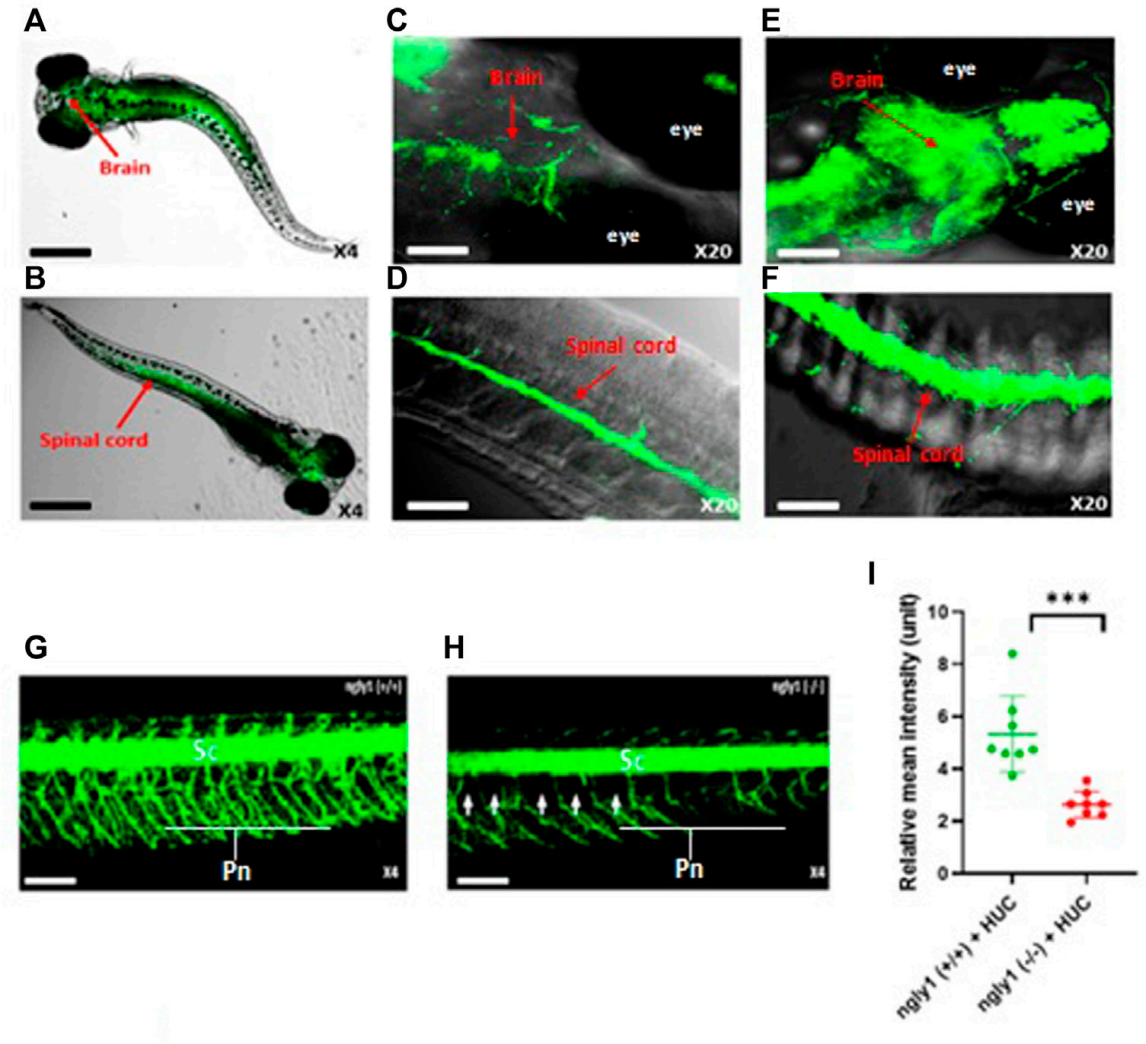
*ngly1* ^(*−/−*)^ larvae displayed loss of axon fibers in the PNS compared to *ngly1* ^(*+/+*)^. **(A)** Dorsal view and **(B)** ventral view **(C)** Brain **(D)** Spinal cord of 6*dpf* larvae (WT) identified by *in situ* hybridization analysis-X4, X20, confocal Nikon eclipse, *n* = 10. **(E)** Brain **(F)** Spinal cord of Tg (HUC:gal4 uas:mem YFP-mito CFP)- X20, confocal Nikon eclipse, *n* = 5. **(G)** *ngly1* ^(*+/+*)^ control and **(H)** *ngly1* ^(*−/−*)^ mutant groups at 6*dpf*. Sc = spinal cord, Pn = Peripheral nerves, white arrow represents the loss of axon fibers in PNS. X4, confocal Nikon eclipse. **(I)** Quantification of fluorescence by neurons, t-test (*n* = 8 in each group ***p < 0.001). Scale bars: black- 50 µm, white- 20 µm.

To study the expression pattern of *ngly1* in the CNS and PNS, we performed *in-situ* hybridization analysis in larvae nervous system. *Ngly1* is significantly expressed in the brain, spinal cord and peripheral nervous system of WT ZF larvae (Figures 4A–D). In order to validated the *ngly1* expression in the nervous system, the green fluorescent regions were compared to a Tg (HUC:gal4 uas:mem YFP-mito CFP)- fish that constantly expresses a fluorescent protein which labels the nervous system as a positive control (Figures 4E,F).

### 3.4 Nervous System Live Imaging Analysis

Nervous system morphology was analyzed at 6*dpf* in *ngly1* ^(*−/−*)^/Tg (HUC:gal4 uas:mem YFP-mito CFP) and *ngly1* ^(*+/+*)^/Tg (HUC:gal4 uas:mem YFP-mito CFP) sibling. The results demonstrate that *ngly1* ^(*−/−*)^ display a significant reduction (*p* < 0.001) of peripheral axon fibers in comparison to control group (Figures 4G–I).

### 3.5 Musculoskeletal Morphology

**FIGURE 5:**
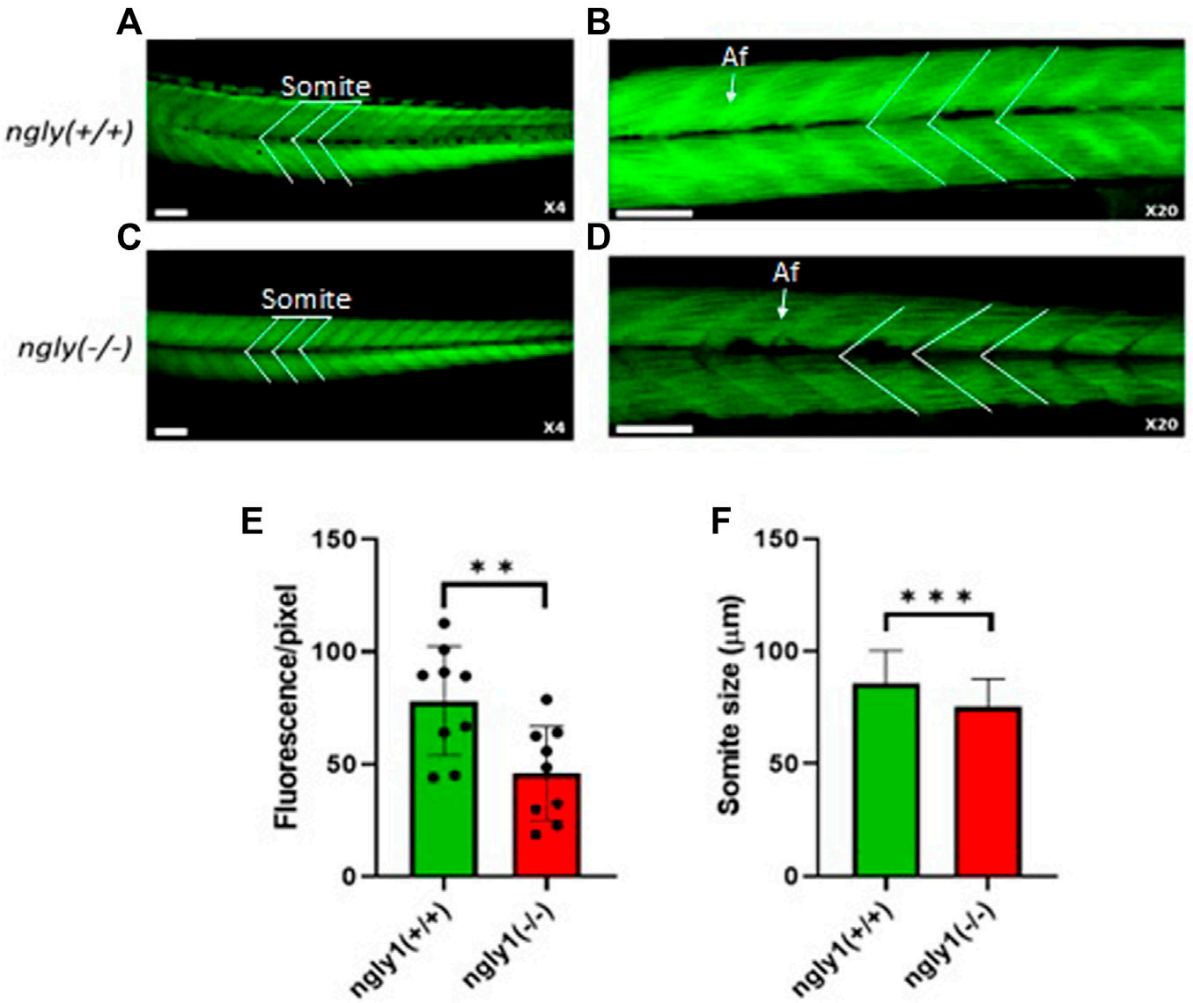
Muscle structure morphology. Representation of *ngly1* ^(*+/+*)^ **(A,B)** and *ngly1* ^(*−/−*)^ **(C,D)** Phalloidin staining for actin (A-X4 and B-X20). Af = Actin filaments. **(E)** Quantification analysis of the Phalloidin fluorescence. t-test (*n* = 9 in each group, **p < 0.01). **(F)** Mean size of somite (µm). t-test (*ngly1* ^(*+/+*)^, *n* = 65. ngly*1* ^(*−/−*)^, *n* = 79, ***p < 0.001). Scale bars—50 µm.

Muscle structure was analyzed at 6*dpf* in *ngly1* ^(*−/−*)^ fish and WT siblings. The result confirmed that *ngly1* ^(*−/−*)^ fish demonstrate a reduction in muscle mass (*p* < 0.01) and somite size (*p* < 0.001) in comparison to the control group (Figure 5).

### 3.6 Motor Assays

**FIGURE 6:**
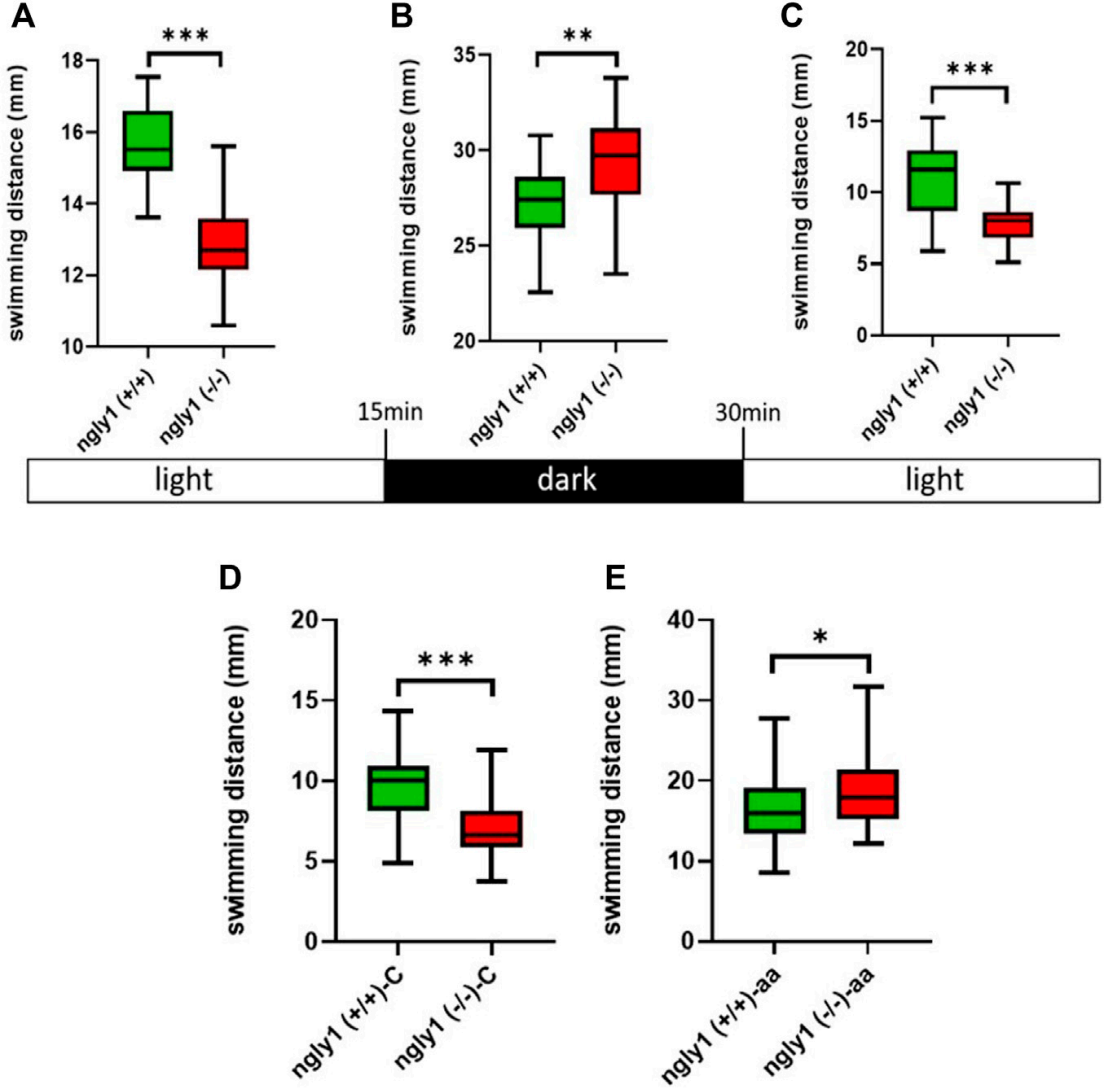
Locomotion analysis. **(A–C)** Quantification of swimming distance of *ngly1* ^(*+/+*)^ and *ngly1* ^(*−/−*)^ larvae for 5 min at various consecutive light conditions: light-dark-light. Each experiment was repeated 3 times, *n* = 91, t-test, **p < 0.01, ***p < 0.001. **(D)** Quantification of swimming distance in larvae (6*dpf*) at normal conditions. C: control, t-test (*n* = 12 in each group, ***p < 0.001). **(E)** Quantification of swimming distance after acetic acid (pH = 3.8) treatment-*ngly1* ^(*+/+*)^ compared to *ngly1* ^(*−/−*)^ from one independent experiment. aa: acetic acid, t-test (*n* = 36 in each group, *p < 0.05).

To investigate the functionality of the MSK system in *ngly1* mutant fish, we tested ZF swimming. The *ngly1* ^(*−/−*)^ larval ZF displayed significantly less swimming distance with respect to the control *ngly1* ^(*+/+*)^ (*p* < 0.001). Under stress stimulus, when lights were turned off, both groups showed an increase in the distance moved compared to lights-on phase (Figures 6A–C). However, *ngly1* ^(*−/−*)^ fish showed a significantly higher increase in swimming distance than the control *ngly1* ^(*+/+*)^ fish (*p* < 0.01)*.*

### 3.7 Sensitized Acid Aversion Assay

To further characterize the neural phenotype, we performed a pain insensitivity test by sensitized acid aversion assay. The premise of this experiment is based on the nociceptive response presented by zebrafish larvae. Steenbergen and Bardine (2014), demonstrated that zebrafish larvae show behavioral responses to a noxious chemical stimulus and that the sensory nervous system receptors mediate a response to pain by acid aversion. The two fish groups—*ngly1* ^(*−/−*)^ and *ngly1* ^(*+/+*)^ were compared at the larval stage (6*dpf*). The *ngly1* ^(*−/−*)^ larvae reacted to the pain with a significant increase in swimming distance compared to *ngly1* ^(*+/+*)^ after the treatment by acetic acid (pH 3.8) (*p* < 0.05) (Figures 6D,E).

## 4 Discussion

In the present study we established a viable *ngly1* mutant zebrafish model and studied the phenotypic consequences of the perturbed *ngly1.* The chosen fish model offers unique opportunities for gene modification by CRISPR/Cas9 technologies followed by gross morphological examination, live imaging of the dynamic behavior and anatomic characterization of the whole organism, from the embryonic to adult stages. As far as we know, no zebrafish model for NGLY1 deficiency had been generated to date.

Our research was based on a study of four patients from two related Druze families who presented with clinical features of *NGLY1* deficiency (Kalfon et al., 2021).

The literature describes several attempts to create animal models that mimic the disease in mice (Fujihira et al., 2017), *Drosophila* (Galeone et al., 2017) and rats (Asahina et al., 2020). However, there was little evidence for the causative role of *NGLY1* disruption in the pathophysiology of the disease.

We created a nonsense genetic variant in exon 9 from 11 exons in a zebrafish model, similar to the genetic variant in our patients that disrupts the normal gene sequence and leads to a premature termination codon in position 534 of the protein (ngly1’s full protein length is 644 amino acid). The AA sequence of the mutant fish is not identical to that of human patients, however it is very similar as both the human and fish mutation are a nonsense mutation in exon 9 from 11 exons. qPCR analysis of mRNA levels in the zebrafish revealed significant reduction in the levels of ngly1 mRNA in the *ngly1* ^(*−/−*)^ fish compared to the *ngly1* ^(*+/+*)^. We also observed massive decrease in the levels of ngly1 protein in the *ngly1* ^(*−/−*)^ fish compared to the *ngly1* ^(*+/+*)^.

However, we demonstrated a residual amount of the ngly1 protein in the mutant fish. Indeed, Smits et al. (2019) described this phenomenon of residual protein expression, that is, characteristic to the CRISPR-Cas9 system, supporting the validity of our data. Another possible explanation for the residual protein in the mutant fish, is alternative splicing variation in different tissues of the fish model. However, since exon 9 of ZF codes for 63 amino acids (∼7 KD) and the WB analysis showed a normal size of the mutant protein, the mechanism of exon skipping is less likely to be the cause for the presence of residual protein. The residual protein in the mutant fish has size similar to the normal ngly1 protein, therefore we assume that there is minimal catalytic activity in the mutant fish. This enzymatic activity may explain the fact that mutant larva present with “mild” phenotype compared to the human patients carrying the same genetic variant.

A number of loss-of-function pathogenic variants in human *NGLY1* have been reported, including nonsense, missense, frameshift, and splice site variants. These occur all through the gene, with no obvious hot spots (Gene reviews NGLY1). The p.Arg401Ter nonsense variant is the most common, accounting for approximately one third of pathogenic alleles (Enns et al., 2014). Affected individuals harboring at least one copy of c.1201A>T (p.Arg401Ter) tend to have a more severe clinical course with higher scores on the Nijmegen Pediatric CDG Severity scale (Lam et al., 2017). A sib pair with the cryptic pathogenic c.930C>T splice site variant (predicted as a silent p.Gly310=) and a p.Gln208Ter nonsense variant exhibited relatively mild impairment in all domains (Lam et al., 2017). Nevertheless, no clear phenotype genotype correlation has yet been established.

In order to compare between the phenotypes of NGLY1 deficiency fish model and the human patients, we explored and characterized two systems: the nervous system and the MSK system.

The expression pattern of ngly1 in ZF tissue has not been reported before. Therefore, we focused on the anatomic regions defined by *ngly1* expression in 6*dpf* larvae by RNA *in situ* hybridization. In the ZF larvae we observed significant expression of *ngly1* mRNA in the nervous system (brain and the spinal cord) as hypothesized (Figures 4A–D), suggesting the importance of ngly1 in ZF nervous system development and maintenance. Furthermore, we demonstrated a distinct neural phenotype of the PNS morphology showing a loss of axon fibers; this finding supports a causative correlation of the NGLY1 nonsense genetic variant and the neurological phenotype in ZF and possibly in humans. Furthermore, we analyzed ZF locomotion behavior during startling by light/dark switch (Hamilton et al., 2017). The general motor test showed a reduction of movement in *ngly1* ^(*−/−*)^ larvae compared to *ngly1* ^(*+/+*)^ with light on (ideal conditions) similar to the phenotype reported in a rat model and in patients who universally present with delayed or lost motor milestones. In contrast, under stress stimulus, when lights were turned off, both groups showed an increase in swimming distance (Figure 6B) compared to lights-on phase (Figures 6A–C). However, *ngly1* ^(*−/−*)^ fish increased the swimming distance significantly compared to the control *ngly1* ^(*+/+*)^ fish. This result revealed a surprising observation, by which *ngly1* ^(*−/−*)^ fish have an impairment of the sensory regulation system—hypersensitivity to a stress trigger unlike the human patients and the rat model. Clinical case reports described pain insensitivity in patients with NGLY1 deficiency. In the case of the ZF model, the *ngly1* ^(*−/−*)^ larvae reacted to a pain trigger with an increase in swimming distance compared to *ngly1* ^(*+/+*)^ after treatment with acetic acid (pH 3.8). Since the increase in fish movement correlates to pain response (Curtright et al., 2015), we conclude that the *ngly1* ^(*−/−*)^ larvae displayed pain hypersensitivity reactions (Figures 6D,E) unlike the human patients and the rat model. This result provides additional evidence of unexpected phenotype of sensory hypersensitivity reaction in junior mutant ZF. Finally, our study shows an increase in the sensitivity of the mutant fish model to stress conditions. The mechanism of this phenomenon is yet to be determined.

We speculate that this phenomenon can be explained by the fact that the loss of peripheral axons is part of complex peripheral neuropathy, which can lead to hyperesthesia. Moreover, in order to add understanding about the mechanism underlying the paradoxical behavior of the mutant fish, it is important to further investigate the fish brain, that is, known to have a crucial role in responding and regulation of stress stimuli (Maldonado and De Jesus, 2021).

Another phenotype found in patients is MSK abnormalities that are described as one of the secondary phenotypes of NGLY1 deficiency and occur as a result of the loss of motor control (Need et al., 2012). In order to explore the MSK system phenotype and understand in which stage of the disease the muscle phenotype starts taking place, we studied the muscle morphology of 6*dpf* larvae. Similar to the human patients (Panneman et al., 2020), we observed a significant difference in the muscle structure and morphology between the two ZF genotypes—*ngly1* ^(*−/−*)^ and *ngly1* ^(*+/+*)^.

In general, the ZF model for NGLY1 deficiency does not display the anticipated dramatic phenotype such as global developmental delay (He et al., 2015). This might be explained by the fact that there is residual protein level in *ngly1* ^(*−/−*)^ fish. Also, another possible explanation is based on the study that has described an effective compensation mechanism in ZF as a response for nonsense-mediated mRNA decay (NMD) in case of premature stop codon in mRNA (El-Brolosy et al*.*, 2019) as we created in our fish model.

In summary, we have created a ZF model for NGLY1 deficiency and characterized a neural phenotype of loss of axon fibers in PNS, a MSK phenotype of reduction in muscle mass and somite size, and a surprising sensory hypersensitivity to external triggers such as darkness and acetic acid-related pain. These multi-organ phenotypes of the mutant fish, suggest—a versatile function of NGLY1—and possible tissue specific mechanisms underlying the complexed phenotype of NGLY1 deficiency. Therefore, future studies should focus on several specific tissues in paralleled to studying the entire fish to accurately unveil the mechanism of this disorder. Further characterization of the brain, liver, skeleton, and eyes in adult fish might enable documentation of the natural history and the progression of the disease in comparison to the human phenotype. Our results further support previous reports showing that zebrafish are highly amenable to mutagenesis and germline transmission. Continued investigations of the NGLY1 deficiency ZF model might lead eventually to resolving the molecular details and mechanisms behind this rare systemic disorder and might serve as a platform for experimental treatments to rescue/medicate this devastating disease in humans.

## Data Availability

The datasets presented in this study can be found in online repositories. The names of the repository/repositories and accession number(s) can be found in the article/supplementary material.
